# Biochar in Co-Contaminated Soil Manipulates Arsenic Solubility and Microbiological Community Structure, and Promotes Organochlorine Degradation

**DOI:** 10.1371/journal.pone.0125393

**Published:** 2015-04-29

**Authors:** Samuel J. Gregory, Christopher W. N. Anderson, Marta Camps-Arbestain, Patrick J. Biggs, Austen R. D. Ganley, Justin M. O’Sullivan, Michael T. McManus

**Affiliations:** 1 New Zealand Biochar Research Centre, Massey University, Palmerston North, New Zealand; 2 Soil and Earth Sciences, Institute of Agriculture and Environment, Massey University, Palmerston North, New Zealand; 3 Institute of Veterinary, Animal and Biomedical Sciences, Massey University, Palmerston North, New Zealand; 4 Institute of Natural and Mathematical Sciences, Massey University, Auckland, New Zealand; 5 Liggins Institute, University of Auckland, Auckland, New Zealand; 6 Institute of Fundamental Sciences, Massey University, Palmerston North, New Zealand; Dowling College, UNITED STATES

## Abstract

We examined the effect of biochar on the water-soluble arsenic (As) concentration and the extent of organochlorine degradation in a co-contaminated historic sheep-dip soil during a 180-d glasshouse incubation experiment. Soil microbial activity, bacterial community and structure diversity were also investigated. Biochar made from willow feedstock (*Salix sp*) was pyrolysed at 350 or 550°C and added to soil at rates of 10 g kg^-1^ and 20 g kg^-1^ (representing 30 t ha^-1^ and 60 t ha^-1^). The isomers of hexachlorocyclohexane (HCH) alpha-HCH and gamma-HCH (lindane), underwent 10-fold and 4-fold reductions in concentration as a function of biochar treatment. Biochar also resulted in a significant reduction in soil DDT levels (P < 0.01), and increased the DDE:DDT ratio. Soil microbial activity was significantly increased (P < 0.01) under all biochar treatments after 60 days of treatment compared to the control. 16S amplicon sequencing revealed that biochar-amended soil contained more members of the *Chryseobacterium*, *Flavobacterium*, *Dyadobacter* and *Pseudomonadaceae* which are known bioremediators of hydrocarbons. We hypothesise that a recorded short-term reduction in the soluble As concentration due to biochar amendment allowed native soil microbial communities to overcome As-related stress. We propose that increased microbiological activity (dehydrogenase activity) due to biochar amendment was responsible for enhanced degradation of organochlorines in the soil. Biochar therefore partially overcame the co-contaminant effect of As, allowing for enhanced natural attenuation of organochlorines in soil.

## Introduction

The rearing and sale of sheep has made a significant contribution to New Zealand’s economy. Throughout much of the 19^th^ and 20^th^ centuries there was a legal requirement that all animals sold were pest free [1], and this was achieved by submerging sheep in pesticide baths containing organochlorines and arsenicals. This practice occurred on every sheep farm in New Zealand between 1840 and 1960 [1–3]. The legacy of this practice today is an estimated 50,000 historic sheep-dip sites throughout New Zealand that potentially pose a risk to the environment.

Organochlorines were more effective than arsenic (As) for the control of parasites and gradually superseded As from the 1940s onwards as awareness of the environmental danger of As increased [1,4]. The chlorinated cyclodienes (aldrin, dieldrin, heptachlor) and the chlorobenzene derivatives of these cyclodienes (dichlorodiphenyltrichloroethane [DDT]; and ϒ-hexachlorocyclohexane [lindane]) were commonly used in pure or mixed forms (*e*.*g*. lindane was often applied as a mixture of HCH isomers [technical HCH]) [5,6]. The use of organochlorines in pest control was subsequently banned when it became clear that they accumulate in body fat and can subsequently be passed onto consumers [3,7].

Organochlorines can naturally degrade in soil through the mechanistic pathways of dechlorination, dehydrochlorination, isomerisation and oxidation [8–10]. Each of these mechanisms is dependant on soil microbial activity, and when this is inhibited so is the potential for natural attenuation in the soil. Inhibition of microbial activity can occur as a result of soil contamination with heavy metals and metalloids [11]. Due to the fact that most historic sheep dip sites can be considered as co-contaminated with organochorines and As, many authors have suggested that co-contamination is detrimentally affecting the makeup and population of microbial communities in these soils, inhibiting the potential for their natural bioremediation [12–14].

The effectiveness of bioremediation can be increased through the addition of specific amendments to soil that stimulate soil microbiological activity [15–17]. One such amendment is biochar [18–20]. Biochar has properties that can affect the retention and release of soil contaminants and nutrients [21–23]. For example, Beesley & Marmiroli [24] reported that biochar could retain As from a leachate solution contaminated with the metalloid. No mechanistic information was provided to explain this retention although this may be related to physical or chemical interactions between As and the biochar surface.

Key to the performance of biochar in soil is the method used to prepare the carbon material. Temperature of production in particular will control the surface area of the final product. For example, biochar produced from woody material at low temperature (e.g. 400°C) tends to have a low surface area (<5 m^2^ g^-1^), whereas that produced from woody material at high temperature (e.g. 550°C) generally has a high surface area (>55 m^2^ g^-1^) [25,26]. Adsorption of hydrophobic organic compounds is expected to be greater to biochars with high surface area (e.g. high temperature biochars) [27–29], although blockage of the pore neck of biochar by adsorbed compounds has been reported, subsequently decreasing the sorption properties of the soil amendment [30]. Low temperature biochars have a greater fraction of volatiles than high temperature biochars, which represents a potential energy and carbon source for soil microbiological activity [31].

Amending a soil with a source of labile carbon has the potential to stimulate microbial activity promoting dechlorination of organochlorines and a subsequent reduction in the half-life of these chemicals in soil [15,32,33]. The addition of a carbonaceous material can also induce the sorption of some organochlorines (pesticides) to soil [29,34,35]. To date, no studies have analysed the specific response of soil microbes to biochar amendment of a soil co-contaminated with organochlorines and As. Our work was therefore designed to test the hypothesis that biochar-amendment to a co-contaminated sheep-dip soil would stimulate the natural bioremediation of organochlorines. We sought to investigate the potential of biochar as a simple and effective soil amendement to effect the remediation of soil contaminants at historic ship-dip sites. The work described in this paper was conducted concurrently with a plant growth trial that recorded a short-term reduction in water-extractable As in soil as a function of the addition of willow biochar to soil, and an increase in soil dehydrogenase activity (DHA) over a 180-d period [36]. This increase in DHA was attributed to the observed reduction in water-extractable As causing the natural soil microbial community to be released from As-related stress.

The specific objectives of the study described in this paper were to investigate if biochar produced from willow at two temperatures of pyrolysis (350 and 550°C) can (i) affect microbial activity and the composition of the bacterial communities present in co-contaminated soil, and (ii) enhance the degradation of organochlorines in soil.

## Materials and Methods

### Sample collection and pre-treatment

**Fig 1:**
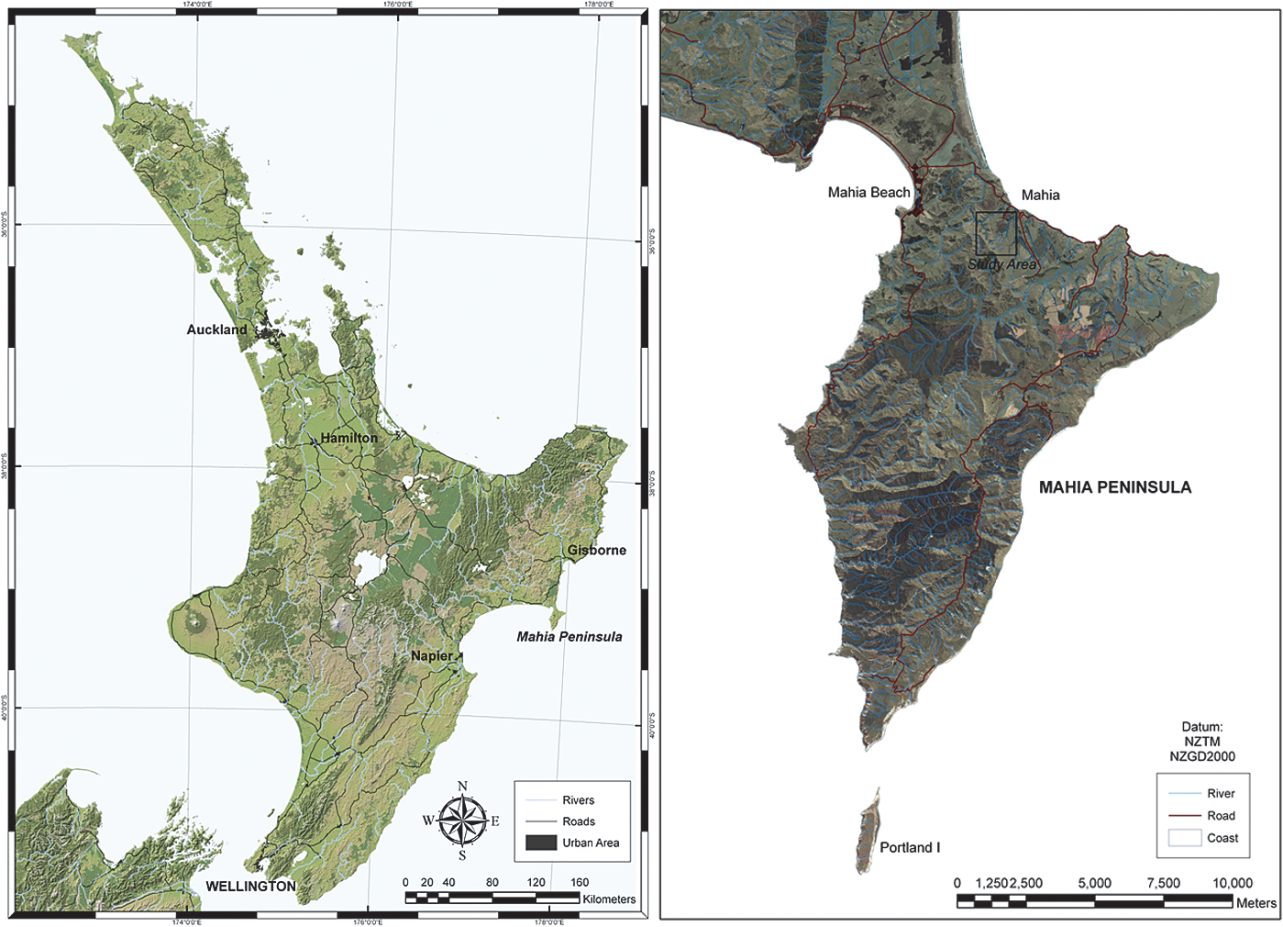
Trial site locality map showing the North Island of New Zealand (left: courtesy of Geographix) and a blow-up view of the site in an area of Te Mahia peninsula on the East Coast (right: courtesy of Land Information New Zealand).

A sheep dip site operational from 1860 to 1980 was identified on private land in Te Mahia, on the East Coast of New Zealand (Fig 1). The soil at this location is a pumice soil (Orthic Allophanic) overlying a marine terrace. A soil survey identified heterogenous arsenic (As) concentrations ranging from 200–2000 mg kg^-1^ in the area directly around the inferred location of the historic dip site (see *Soil and Biochar Analysis*). Organochlorines, including isomers of HCH, aldrin, dieldrin and DDT, were also present at high concentrations. This site characterisation was conducted with the permission of the private landowner. Further information can be obtained from the corresponding author. Subsequent to the reconnaissance work, a bulk soil sample (300 kg) was collected to a depth of 20 cm from a 2 m x 2 m area where residual dip solution was disposed of at the end of each dipping event. After transport to Massey University, the soil was mixed using a nursery grade mixer to homogenise As and organochlorine contamination. The mixed soil was sieved through a 5 mm-mesh to remove coarse inorganic and organic material and stored at 4°C until use.

### Biochar production

Wood from 1- and 5-year-old willow (*Salix* sp.), was collected, chipped and used as a composite feedstock for biochar production according to the method described by Gregory et al. [36]. Two biochars were generated; a high-temperature biochar produced at 550°C and a low-temperature biochar produced at 350°C. Important physico-chemical properties of these biochars are summarised in Table 1.

**Table 1:** Select physio-chemical properties of the willow biochars used in this study (after Gregory et al [36]).

Parameter	350°C Biochar	550°C Biochar
**pH**	8.6	8.6
**C content (g kg** ^**-1**^ **)**	739	774
**N content (g kg** ^**-1**^ **)**	8.0	7.0
**H content (g kg** ^**-1**^ **)**	35	33
**Ash content (%)**	5.6	4.1
**Surface area (m** ^**2**^ **g** ^**-1**^ **)**	6.2	59.8
**1 M HCl-extractable K (g kg** ^**-1**^ **)**	11.9	10.7
**1 M HCl-extractable Ca (g kg** ^**-1**^ **)**	23.6	24.0
**1 M HCl-extractable Mg (g kg** ^**-1**^ **)**	1.5	1.4
**1 M HCl-extractable SO**_**4**_ **-S (g kg** ^**-1**^ **)**	1.9	1.8
**2% formic acid-extractable P (g kg** ^**-1**^ **)**	0.7	0.8

### Incubation experiment

Experiments were conducted under greenhouse conditions in freely draining square plastic pots (20 x 20 cm and 30 cm deep). Fertility analysis of the collected soil showed nutrient parameters within agronomic guidelines, and therefore fertiliser (N:P:K) was not applied [37,38].

Two doses of biochar were selected: 10 and 20 g biochar kg^-1^ soil (1% and 2%). When incorporated to 30 cm depth (assuming a soil bulk density of 1 g cm^-3^) this loading corresponds to 30 t ha^-1^ and 60 t ha^-1^, respectively. Sheep dip sites are commonly small (30 m^2^) but highly contaminated, and are amenable to high rates of biochar application. Biochar was manually mixed with soil prior to filling the plastic pots. All pots were maintained at 70% water holding capacity (WHC) by the addition of distilled water. The incubation experiment was conducted over 6 months with three replicate pots per treatment (15 pots in total). Contaminated soil that was not amended with biochar was the control treatment in this study and also maintained at 70% WHC.

Monthly soil samples were collected from every pot for chemical and biological analysis starting 30 days after pot filling (T = 30 d). Samples were taken using a stainless steel corer with an internal diameter of 1 cm. Soil cores were analysed for microbial activity and water-extractable As. To achieve this, at each sampling time, two vertical cores were taken (each core 6 g) and combined in a plastic bag, homogenised, and then split. One half was analysed for microbial activity (fresh soil) and the other for water-extractable As concentration (air-dried soil). Following sampling, cored holes were filled with initial (control) soil and marked with a toothpick, to prevent re-sampling of the same site. New sampling locations were located ~3 cm adjacent to the previous site, at each subsequent sampling time.

### Soil and biochar analysis

#### Biochar

Biochar analysis and properties are described in Gregory et al [36] and Table 1. Briefly, carbon, hydrogen and nitrogen concentrations in the two biochar samples were determined using Elementar vario MACRO CUBE (Elementar; Hanau, Germany). Biochar pH was measured in deionized water at a 1:100 (w/w) ratio according to the method of Ahmedna et al [39] after heating for 20 min at 90°C and cooling to room temperature. The ash content of the biochar samples was determined by thermal analysis using a thermogravimetric analyser (SDT Q600, TA Instruments, Melbourne, Australia). The specific surface area of each biochar was calculated from N_2_ physisorption data according to the Brunauer-Emmett-Teller (BET) method using P/Po values in the range 0.05–0.2 N_2_. Physisorption measurements were performed at -195°C in liquid nitrogen) using a Micromeritics Tristar 3000 instrument. Samples were degassed at 300°C in N_2_ for 4 h prior to the N_2_ adsorption measurements. Extractable P in biochar was estimated using 2% formic acid according to Wang et al [40]. Extractable K, Mg, Ca and SO_4_-S were analysed according to the methods of Blakemore et al [41] with modification. Briefly 0.35 g of finely ground biochar was added to 35 mL 1 M HCl acid solution and placed in 35 mL centrifuge tubes on an over-end shaker overnight. Samples were then filtered and the concentration determined by atomic absorption spectroscopy (AAS) (auto-analyser for SO_4_-S).

#### Soil

Soil pH was measured in deionised water at a ratio of 1:2.5. Available phosphate (Olsen P), sulphate and soil cations were measured according to the methods of Blakemore et al [41] (at T = 0 and 180 d). Available phosphate (Olsen P) was extracted in 0.5 M sodium bicarbonate (1:20 soil:solution ratio) and determined colorimetrically. Available sulphate was extracted with 0.1 M potassium phosphate (1:5 soil:solution ratio) and determined by automated colorimetric technique. Cations were extracted by leaching with 1 M ammonium acetate (pH 7; 1:50 soil:solution ratio) and determined by atomic absorption spectroscopy (AAS). Cation exchange capacity was determined by the summation of extractable cations and the extractable acidity [42]. Important chemical properties of the soil are as follows: pH 5.6; CEC 12 meq/100g; Olsen P 49 μg P g^-1^; sulphate-S 13.4 μg S g^-1^; extractable K^+^ 0.8 me 100g^-1^; extractable Ca^2+^ 3.3 me 100g^-1^; extractable Mg 1.4 me 100g^-1^. Additional soil properties including those of soil amended with biochars are described in Gregory et al [36].

To determine the total As concentration in soil, a sub-sample of homogenised soil (1 g) was first pre-digested in aqua regia (3:1 mixture of concentrated HCl and HNO_3_) overnight, and digested on a heat block (120°C) for 2 hours the following day. Once cool each digest solution was filtered (Whatman 42C) and then made to a final volume of 100 mL with deionised water. To determine the water-extraction concentration of As in each soil, a modified version of the extraction described by Ko et al [43] was used. A sub sample of soil (2 g) was extracted with 20 mL of deionised water in a 25 mL polycarbonate extraction tube (16 hours) using an end-over-end rotating platform. Tubes were then centrifuged at 10 000 x g for 3 min and finally filtered (Whatman 42C). For instrumental analysis using a Flow Injection Analysis System 400 (FIAS, Perkin Elmer) coupled to a Graphite Furnace Atomic Absorption Spectrophotometer (GFAAS; AAnalyst 600, Perkin Elmer). Aliquots of the acid-digest and water-extract solutions were pre-reduced using 5% (w/v) potassium iodide + 5% (w/v) ascorbic acid (1 mL sample + 1 mL HCl + 1 mL reductant). Pre-reduced samples were left to stand for 45 min and then diluted to 10 mL with 10% HCl for quantification of the As concentration. Working standards were prepared from the As (V) salt Na_2_HAsO_4_.7H_2_O and a minimum correlation coefficient for the standard curve of 0.995 was required before analysis could proceed. A certified soil reference CRM—GBW 07403 (National Research Centre for CRMs of China) was used to confirm the accuracy of the soil arsenic measurements. Analysed As values for the soil reference material differed by < 10% from the certified mean concentration. The total As concentration of the soil was 202 (± 3.9) mg kg^-1^.

### Dehydrogenase activity (DHA) assay

Microbial oxidation of organic compounds is directly linked to the electron transport chain (ETC) that utilises oxygen as a final electron acceptor. Dehydrogenases are fundamental enzymes that form a critical part of the microbial ETC [44]. Dehydrogenase activity can be empirically measured using artificial electron acceptors like tetrazolium salt. Thus, DHA is a good indicator of total microbial activity. In the current work, we quantified microbial activity by measuring the DHA in the soil according to the method of Chander & Brookes [45] with modification. Briefly, the DHA in 5 g of fresh soil was activated by the addition of 0.1 g CaCO_3_. Three mL of tetrazolium chloride (TTC) was added to the activated soil, in the absence of light, and the resulting mixture vortexed to remove any trapped air spaces. The mixture subsequently incubated (28°C, overnight) and the resulting tri-phenyl formazan (TPF) extracted with 20 mL methanol. Colorimetric intensity was measured at 490 nm using a Jenway Spectrophotometer (Jenway 7315) to determine formazan concentration. Standard curves were constructed for each treatment to limit the effect of TPF colour absorption caused by biochar.

### Organochlorine analysis

Organochlorine type and concentration in soil samples were analysed by an international accredited laboratory [38]. Fresh soil cores were taken at T = 180 d using a 10 cm steel corer from undisturbed regions within each pot, placed in sealed glass jars and stored at 4°C prior to analysis. Organochlorines were extracted using a hexane-acetone sonication extraction. Phosphoric acid was utilised as a pre-wetting step to expand the soil matrix before full extraction using Gas Chromatography-Mass Spectrometry (GC-MS).

### Soil bacterial community structure and diversity

At the conclusion of the incubation experiment (T = 180 d), soil cores were extracted from undisturbed regions within each pot using a steel corer (1 cm diameter, 10 cm deep). Three cores were extracted from each control and high dose biochar pot (60 t ha^-1^) and combined. Between each treatment core extraction, the corer was thoroughly rinsed in 70% EtOH (ethanol) and dried to prevent cross-contamination. Each core was placed in sterile 15 mL falcon tubes and stored at 4°C prior to DNA extraction.

#### Sample extraction for 16S amplicon analysis

Total DNA was extracted from 0.25 g of soil from each treatment, in triplicate, using the Powersoil DNA extraction kit according to the manufacturer’s instructions (Mo Bio Laboratories). Following elution, DNA was stored at -80°C until use.

#### PCR amplification

PCR was performed using SRV3 universal primers (SRV3-1/SRV3-2) [46] which amplify the V3 region of the 16S ribosomal subunit (rDNA). Primers had 5’ extensions with unique Molecular Identifier (MID) tags (Table 2). Two independent PCR reactions were performed using 2 μl of extracted DNA, 1 X high fidelity PCR master mix (Roche Cat. No. 12 140 314 001) and 0.5 mM SRV3 primers for each sample. Amplification conditions were: ((1x 95°C, 3min); (35 x (95°C, 30sec; 52°C 30sec; 73°C, 30sec)); (1x 72°C, 30min); (1x 4°C, ∞)). Following PCR, the two independent PCR reactions for each sample were pooled prior to AMPure purification.

**Table 2:** Primer sequences used in this study. MID tag numbers with associated treatment are in italics. Primers had 5’ extensions with unique tags to identify correct DNA sequence.

Sample	Primer name	MID sequence	MID+SRV3 (forward/Reverse)	SRV3 primer
Control	SRV FWD-52/1-125	ATGTACGATG	*ATGTACGATG*CGGYCCAGACTCCTACGGG	CGGYCCAGACTCCTACGGG
	SRV REV-52/1-125	ATGTACGATG	*ATGTACGATG*TTACCGCGGCTGCTGGCAC	TTACCGCGGCTGCTGGCAC
Control	SRV FWD-52/3-127	CACACGATAG	*CACACGATAG*CGGYCCAGACTCCTACGGG	CGGYCCAGACTCCTACGGG
	SRV REV-52/3-127	CACACGATAG	*CACACGATAG*TTACCGCGGCTGCTGGCAC	TTACCGCGGCTGCTGGCAC
Control	SRV FWD-52/4-128	CACTCGCACG	*CACTCGCACG*CGGYCCAGACTCCTACGGG	CGGYCCAGACTCCTACGGG
	SRV REV-52/4-128	CACTCGCACG	*CACTCGCACG*TTACCGCGGCTGCTGGCAC	TTACCGCGGCTGCTGGCAC
550 BC	SRV FWD-53/1-130	CAGTACTGCG	*CAGTACTGCG*CGGYCCAGACTCCTACGGG	CGGYCCAGACTCCTACGGG
	SRV REV-53/1-130	CAGTACTGCG	*CAGTACTGCG*TTACCGCGGCTGCTGGCAC	TTACCGCGGCTGCTGGCAC
550 BC	SRV FWD-53/2-132	CGATCTGTCG	*CGATCTGTCG*CGGYCCAGACTCCTACGGG	CGGYCCAGACTCCTACGGG
	SRV REV-53/2-132	CGATCTGTCG	*CGATCTGTCG*TTACCGCGGCTGCTGGCAC	TTACCGCGGCTGCTGGCAC
550 BC	SRV FWD-54/1-135	CGTGATGACG	*CGTGATGACG*CGGYCCAGACTCCTACGGG	CGGYCCAGACTCCTACGGG
	SRV REV-54/1-135	CGTGATGACG	*CGTGATGACG*TTACCGCGGCTGCTGGCAC	TTACCGCGGCTGCTGGCAC
350 BC	SRV FWD-54/2-136	CTATGTACAG	*CTATGTACAG*CGGYCCAGACTCCTACGGG	CGGYCCAGACTCCTACGGG
	SRV REV-54/2-136	CTATGTACAG	*CTATGTACAG*TTACCGCGGCTGCTGGCAC	TTACCGCGGCTGCTGGCAC
350 BC	SRV FWD-54/3-137	CTCGATATAG	*CTCGATATAG*CGGYCCAGACTCCTACGGG	CGGYCCAGACTCCTACGGG
	SRV REV-54/3-137	CTCGATATAG	*CTCGATATAG*TTACCGCGGCTGCTGGCAC	TTACCGCGGCTGCTGGCAC
350 BC	SRV FWD-54/4-139	CTGCGTCACG	*CTGCGTCACG*CGGYCCAGACTCCTACGGG	CGGYCCAGACTCCTACGGG
	SRV REV-54/4-139	CTGCGTCACG	*CTGCGTCACG*TTACCGCGGCTGCTGGCAC	TTACCGCGGCTGCTGGCAC

#### Sequencing preparation

SRV3 16S PCR amplicons were purified using Agencourt AMPure magnetic beads (Beckman Coulter), assayed for purity and quantified using a nanodrop. Amplicons were pooled together in equal amounts (119 ng) and sent for sequencing (Massey Genome Service). Pooled amplicons were then distributed amongst six paired-end sequencing runs (of 250 bp) performed using an Illumina MiSeq (Massey Genome Service, New Zealand Genomics Limited). PhiX174 was used as a sequencing control.

#### Sequence analysis

MiSeq reads were initially processed for quality using custom Perl scripts on a run by run basis. The sequences were analysed with SolexaQA [47] to obtain an overall quality of the runs. The sequences were analysed to remove any PhiX174 reads by using Bowtie2 to map to the PhiX174 genome to create SAM (Sequence Alignment/Map Format) (http://samtools.sourceforge.net/SAMv1.pdf) files. The sam2fastq tool within Picard (http://picard.sourceforge.net/) was used to recreate two Fastq files for each read. Length adjustment of the short reads on a per-read basis was performed using FLASh (http://ccb.jhu.ed/software/FLASH) [48]. Reads were then split into nine files (three for each of the three treatments) according to their MID using fastx_barcode_splitter.pl (part of the fastx toolkit; http://hannonlab.cshl.edu/fastx_toolkit/). Reads were subjected to a second round of quality trimming using DynamicTrim (a part of SolexaQA) at and an error probability cutoff of 0.05 to remove any FLASh introduced anomalies. For a given MID the reads from each of the 6 runs were then combined randomly to generate a concatenated file. Two separate sets of analyses were performed on these files. In the first, a random set of 6,000 reads were taken from each of the concatenated files to investigate the sample variation. In the second, files containing 100,000 trimmed reads were chosen randomly from the pooled sequences for each sample. Both sequence datasets were formatted by custom Perl scripts to provide the correct input fasta format for QIIME v1.6 (Quantitative Insights into Microbial Ecology) [49].

These fasta-formatted reads were analysed in QIIME v1.6 using default parameters [49]. Briefly, sequences were assigned to operational taxonomic units (OTUs) using uclust [50] at 97% similarity. Representative sequences were chosen for each OTU [49]. The representative sequence set was aligned to the Greengenes Core reference alignment [51] using PyNAST [52]. The default taxonomy assignment was performed using RDP Classifier 2.2 [53], and the Greengenes taxonomy and Greengenes reference database [54,55]. The alignments were filtered prior to tree building to remove gaps (filter_alignment.py), An approximately-maximum-likelihood phylogenetic tree was generated using FastTree 2.1.3 [56]. The number of times each OUT was found in each sample was tabulated using make_otu_table.py [49].

Alpha diversity was measured using default parameters and visualized as rarefaction plots. Both weighted and unweighted unifrac measures of the beta diversity were calculated using default parameters [57].

Sequence information has been deposited in the Sequence Read Archive (SRP051260) and under Bioproject PRJNA270654 at the National Center for Biotechnology Information (NCBI).

### Statistical analysis

Three replicates were used for all treatments. Statistical differences between treatments were determined by analysis variance (ANOVA) using SPSS software version 16.0 (SPSS Inc., Chicago, USA).

### Potential of soil dilution by biochar

Data was corrected to avoid the dilution effect of biochar in final concentrations of the different parameters analysed. This was done by multiplying the amount of biochar added, in this case 1 and 2%, to obtain final concentrations. Here, final concentrations were multiplied by 100 and divided by 99 for the (1%) and for 2% biochar multiplied by 100 and divided by 98.

## Results

### Concentration of organochlorines in soil at the end of experiment

**Fig 2:**
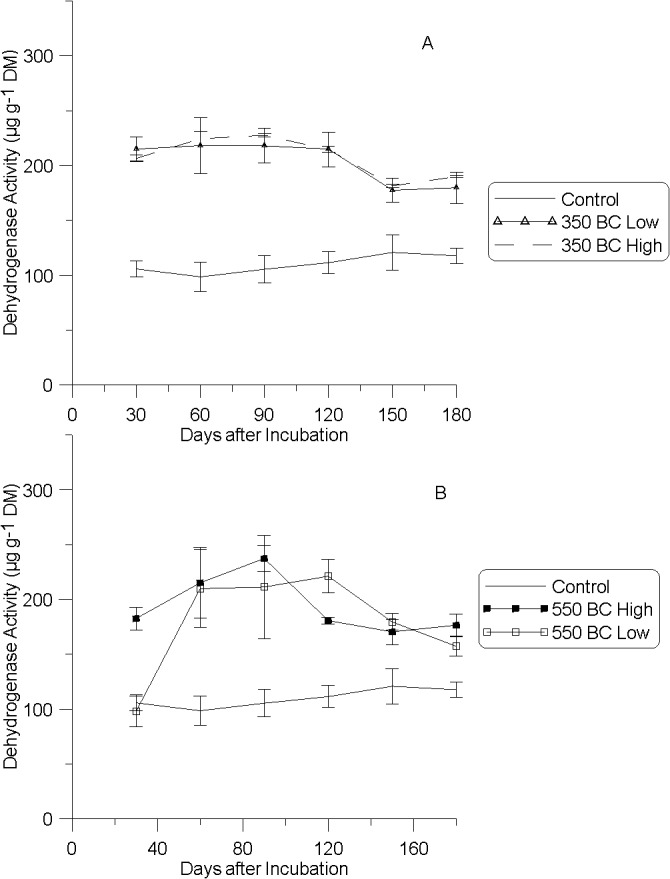
Soil concentrations (mg kg^-1^) for A) Alpha-HCH B) Lindane C) DDT and D) Delta-HCH at the termination of the glasshouse trial under different treatments. Significant differences are observed between all biochar types and the control (P<0.01) (mean *n* = 3; ± s.e.).

Additions of both types of biochar at both rates caused a significant decrease in the soil concentration of two isomers of HCH: alpha-HCH (Fig 2A) and gamma-HCH (Fig 2B) (P < 0.01) (Table 3). Alpha HCH and gamma-HCH concentrations underwent ~10-and 4-fold reductions, respectively, under all biochar treatments. There was no significant difference (P > 0.05) in the extent of reduction between the four types of biochars studied. DDT (including its breakdown products: sum DDT) levels were significantly reduced (P < 0.01) by three of the four treatments under study (350°C biochar at 1 and 2% and also 550°C biochar at 2%; Fig 2C) (Table 3). The magnitude of the decrease in DDT concentration was influenced by the biochar treatment used. 350°C biochar at 2% resulted in a reduction of 38% of total DDT in soil compared to the control, while 350°C biochar (1%) and 550°C biochar (2%) reduced total DDT concentrations by 27 and 25%, respectively. There was also a small but significant reduction (P < 0.05) in delta HCH concentrations following treatment with biochar (Fig 2D) (Table 3). In contrast, beta HCH was unaffected by biochar amendment after 6 months of incubation in the presence of biochar (P > 0.05).

**Table 3:** Organochlorine concentrations (mg kg^-1^) in soil subject to the treatments of this study.

	Aldrin	Dieldrin	α-HCH	δ-HCH	Lindane	Endrin ketone	∑DDT
**Control Soil**	6.17^a^ ±0.37	111^a^ ± 4.04	25^a^ ± 1.15	1.8^a^ ± 0.06	1.6^a^ ± 0.07	1.8^a^ ± 0.08	6.3^a^
**350°C BC (1%)**	7.6^a^ ± 0.25	117^a^ ± 3.84	2.6^b^ ± 0.31	1.6^b^ ± 0.04	0.5^b^ ± 0.06	1.4^b^ ± 0.05	4.6^b^
**350°C BC (2%)**	9.9^b^ ± 0.52	152^b^ ± 4.84	2.2^b^ ± 0.06	1.5^b^ ± 0.03	0.4^b^ ± 0.01	1.7^a^ ± 0.03	3.9^b^
**550°C BC (1%)**	8.4^b^ ± 0.59	134^b^ ± 8.50	3.8^b^ ± 1.19	1.6^b^ ± 0.05	0.5^b^ ± 0.01	1.5^b^ ± 0.03	6.0^a^
**550°C BC (2%)**	6.1^a^ ± 0.70	106^a^ ± 2.52	2.4^b^ ± 0.40	1.6^b^ ± 0.04	0.4^b^ ± 0.02	1.4^b^ ± 0.04	4.7^b^

Different letters corresponding to each organochlorine represent significant differences. Significant differences are noted at P<0.05 (aldrin, dieldrin, δ-HCH, endrin ketone) and P<0.01 (α-HCH, Lindane, ∑DDT) and are noted by different letters (mean *n* = 3; ± s.e.).

### Total and water-soluble As concentration in soil

There was no change in the total As concentration in soil as a function of treatment at any time during the experiment (data not shown). There was, however, a significant short-term reduction in the concentration of water-soluble As concentration for all biochar treatments 30 days after biochar incorporation into the soil (Table 4). This result mirrors that reported by Gregory et al [36] for an experiment where the same soil was treated with biochar and planted with rye grass. The similarity between the two sets of water-soluble As analysis on the same soil infers that biochar is the key variable that affects the water-extractability of As in soil. Plants have no apparent effect on the concentration of water-soluble As; an observation that is in contrast to the results observed by Ko et al [43] who reported that *Brassica juncea* could increase the concentration of water-soluble As present within As-contaminated gold mine tailings.

**Table 4:** Water extractable As (mg L^-1^) in soil under biochar and control treatments at time (T) in days.

	T = 0 d	T = 30 d	T = 60 d	T = 90 d	T = 120 d
**Control Soil**	1.3 ± 0.14^a^ (*5*.*8*)	1.4 ± 0.10^a^ (*5*.*8*)	1.2 ± 0.08^a^ (*5*.*7*)	1.3 ± 0.04^a^ (*5*.*8*)	1.4 ± 0.01^a^ (*5*.*8*)
**350°C BC (1%)**	1.4 ± 0.12^a^ (*6*.*1*)	0.8 ± 0.00^b^ (*6*.*0*)	1.1 ± 0.04^a^ (*5*.*8*)	1.2 ± 0.02^a^ (*5*.*9*)	1.4 ± 0.05^a^ (*5*.*9*)
**350°C BC (2%)**	1.2 ± 0.18^a^ (*6*.*2*)	0.9 ± 0.05^b^ (*6*.*1*)	1.1 ± 0.03^a^ (*6*.*0*)	1.2 ± 0.02^a^ (*5*.*9*)	1.4 ± 0.04^a^ (*6*.*1*)
**550°C BC (1%)**	1.3 ± 0.08^a^ *(6*.*1)*	0.9 ± 0.04^b^ *(5*.*9)*	1.2 ± 0.00^a^ *(5*.*9)*	1.1 ± 0.07^a^ *(5*.*8)*	1.3 ± 0.08^a^ *(5*.*9)*
**550°C BC (2%)**	1.4 ± 0.14^a^ *(6*.*2)*	1.0 ± 0.11^b^ *(6*.*1)*	1.1 ± 0.07^a^ *(5*.*9)*	1.3 ± 0.16^a^ *(5*.*8)*	1.5 ± 0.02^a^ *(6*.*0)*

Significant differences are denoted by different letters at P<0.05 (mean *n* = 3; ± s.e.). Soil pH values are listed in italics and in brackets for each treatment.

### Dehydrogenase activity (DHA)

**Fig 3:**
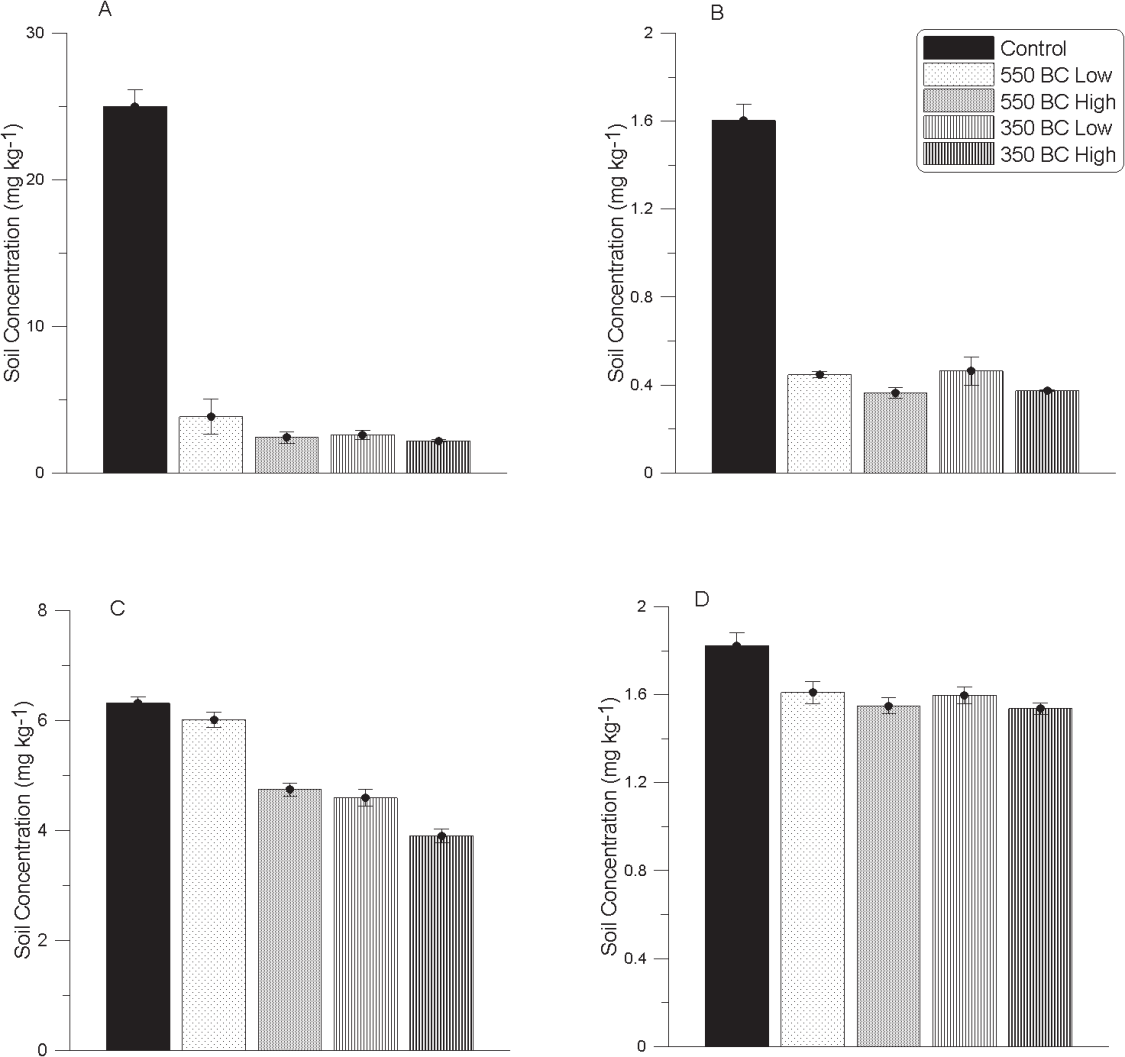
Soil dehydrogenase activity measured in μg per g of dry matter (DM) as a function of A) 350°C biochar treatment and B) 550°C biochar treatment (mean *n* = 3; ± s.e.).

Dehydrogenase activity in the control soil did not change over the six months of the trial, remaining at 100 μg g^-1^ DM (Fig 3A). Following the addition of 350°C biochar, DHA significantly and immediately increased (P < 0.01) at both concentrations (1 and 2%) at every sampling point relative to the control, although there was a general trend to decreasing activity with time. Following the addition of 550°C biochar, DHA was significantly increased at both dosage rates at all sampling times from one-month after biochar incorporation into the soil (Fig 3B). The increase in DHA was further delayed for soil augmented with 1% biochar relative to the 2% treatment. Again there was a noticeable time-dependent decrease in DHA for soil augmented with the 550°C biochar.

### Biochar amendment of soil and its effect on microbial composition

Analysis of the 16S amplicons identified 12 named phyla (and 2 unknown phyla) that contribute significantly to the composition of the bacterial community in all analysed samples (Table 5). Biome specific taxonomic differences were present between each sample (S1 Fig, S2 Fig). Shannon diversity collector’s curves reached saturation for all samples (S3 Fig). The weighted unifrac measure of beta-diversity, which reveals changes due to relative taxon abundance [58], shows that there is a degree of overlap within the samples (S4 Fig) but that biochar treatment correlated with a shift in the population structure (S5 Fig).

**Table 5:** Taxonomy summary of the soil bacterial communities.

	Control	550 BC (2%)	350 BC (2%)
***Actinobacteria***	34.9 ± 2.1	31.3 ± 1.7	**28.0 ± 1.1** ^**a**^
**Other**	17.3 ± 0.0	16.9 ± 0.1	18.3 ± 2.9
***Proteobacteria***	17.7 ± 2.6	**14.2 ± 0.3** ^**a**^	19.9 ± 2.4
***Firmicutes***	9.9 ± 0.2	**12.8 ± 0.9** ^**a**^	9.2 ± 0.4
***Bacteroidetes***	3.2 ± 0.5	3.1 ± 0.8	**6.9 ± 1.3** ^**a**^
***Acidobacteria***	5.0 ± 0.3	5.2 ± 0.3	4.8 ± 0.5
***Planctomycetes***	2.5 ± 0.1	**3.9 ± 0.4** ^**a**^	**3.3 ± 0.1** ^**a**^
***Verrucomicrobia***	2.4 ± 0.2	3.1 ± 0.4	2.9 ± 0.2
***Chloroflexi***	1.9 ± 0.3	2.1 ± 0.3	1.7 ± 0.1
***Gemmatimonadetes***	1.8 ± 0.1	2.0 ± 0.1	1.8 ± 0.2
***Cyanobacteria***	0.4 ± 0.1	2.3 ± 2.0	0.5 ± 0.2
***Nitrospirae***	0.7 ± 0.1	0.8 ± 0.1	0.8 ± 0.1
***Chlamydiae***	0.2 ± 0.0	0.2 ± 0.0	0.2 ± 0.0
**Unknown**	2.1 ± 0.2	2.0 ± 0.0	1.7 ± 0.2

Values are % of population +/- S.E. derived from the taxonomic summaries of the three different biological replicates. Taxonomy is listed based on Phyla (Kingdom: Bacteria) under biochar amendment and no biochar amendment (control). Significant differences are noted by different letters (mean *n* = 3; ± s.e.) compared to the control.

Members of the *Actinobacteria* (31.4%), *Proteobacteria* (17.3%), *Firmicutes* (10.6%), *Acidobacteria* (5.0%) and *Bacteroidetes* (4.4%) dominated the bacterial community structure across all treatments (mean % across all three treatment groups).

Amendment of soil with both biochars (2%) caused a significant shift (P < 0.05) in the relative percentages of several bacterial phyla after 180 d of treatment (Table 5). For the 350°C biochar amendment of soil, this shift consisted of a decrease in the number of *Actinobacteria* and increases in the numbers of the *Bacteroidetes* and *Planctomycetes*. By contrast, there was an increase in the number of *Firmicutes* and *Planctomycetes* and decrease in the number of *Proteobacteria* following the 550°C biochar amendment.

At the genus level a pronounced shift was observed in a number of phyla for the 350°C biochar amendment (Table 6). Within the *Bacteriodetes* a significant (P < 0.05) 3-fold increase in *Chryseobacterium* and *Flavobacterium* was observed along with a 2-fold increase in *Dyadobacter*. Similarly, there was a 2-fold increase in the family *Pseudomonadaceae* (phylum: *Proteobacteria*) when compared to the 550°C biochar amended soil. For 550°C biochar amendment of soil a significant (P < 0.05) 10-fold increase in the order *Streptophyta* (phylum: *Cyanobacteria*) was evident along with small increases in the genus *Bacillus* (phylum: *Firmicutes*), although this result was not significant (P > 0.05).

**Table 6:** Taxonomic structure of the soil bacterial community (%) highlighting genera, families, and orders that showed significant changes between the biochar treatments.

	Control	550 BC (2%)	350 BC (2%)
**(genus) *Chryseobacterium***	0.0 ± 0.0	0.0 ± 0.0	**1.5 ± 0.8** ^**a**^
**(genus) *Flavobacterium***	0.5 ± 0.1	0.3 ± 0.1	**1.2 ± 0.5** ^**a**^
**(genus) *Dyadobacter***	0.5 ± 0.3	0.1 ± 0.0	**1.0 ± 0.1** ^**a**^
**(family) *Pseudomonadaceae***	3.0 ± 1.1^a^	0.8 ± 0.1	**4.8 ± 0.8** ^**a**^
**(order) *Streptophyta***	0.2 ± 0.0	**2.1 ± 0.5** ^**a**^	0.3 ± 0.2
**(genus) *Bacillus***	5.5 ± 0.5	7.1 ± 1.8	4.9 ± 0.1

Values are % of population +/- S.E. derived from the taxonomic summaries of the three different biological replicates. Selected taxonomy is based on significant changes within phyla. Biochar amendment and no biochar amendment (control). Significant differences are noted by different letters (mean *n* = 3; ± s.e.).

## Discussion

### Stimulation of microbial activity with biochar amendment

Dehydrogenase activity is an index of soil microbial activity and has been used to assess the environmental toxicity of chemicals and heavy metals in contaminated soils [45]. Low DHA is common in co-contaminated soils as microbial populations are often supressed by the contaminants, especially As and selected organochlorines [3,10]. Amending soil with a product able to enhance microbial growth and/or diversity may restore microbial communities to uncontaminated levels due to amelioration of the effects of the contaminants on soil microbiology [59,60]. We believe that the initial reduction in water-extractable As (Table 4) in biochar-amended soils may have released soil microbes from As-related toxicity, and combined with a biochar source of labile carbon, may have stimulated microbial activity. The greater efficacy of low-temperature biochar to increase DHA may be attributed to a higher volatile fraction (labile carbon) in this treatment to stimulate microbial activity relative to the high-temperature biochar. However, the possibility of biochar differentially varying soil physiochemical properties (alteration of soil redox potential, water holding capacity, soil pH) cannot be discounted as a factor in the increased microbial activity [15,36,61–63].

Soil microbial activity and a shift in microbial community structure has been observed in soils amended with differing biochars [19,32,64], yet this effect has not been studied in a co-contaminated soil. Warnock et al [31] suggested that biochar with high microporosity forms refuges for microbes when added to soil. The 550°C biochar had a greater surface area (59.8 m^2^ g^-1^) relative to the 350°C biochar (6.1 m^2^ g^-1^) (Table 1), but the effect of this on microbial activity is unclear, as this surface area is mostly associated to nanopores to which soil microbes have no access.

Gamma and alpha-HCH decreased in concentration and DDT and its breakdown derivatives also exhibited reductions after 180 d of biochar amendment (Fig 2; Table 3). Associated with these decreases was a significant increase in DHA that we have used as an index of microbial activity. The bacterial phyla composition we observed at the end of the experiment is similar to the community observed by Sutton et al [65] at a diesel-contaminated railway site where biodegradation of organochlorines was apparent (relatively high abundances of *Proteobacteria*, *Firmicutes*, *Actinobacteria*, *Acidobacteria* and *Chloroflexi*).

Microbial degradation of organochlorines occurs via four main pathways [8,66]: reductive dechlorination, dehydrochlorination, oxidation and isomerisation. Each of these pathways can occur through soil microbiological activity. However, different species and densities of microorganisms will lead to the degradation of organochlorines at different rates. For example, the yeast (fungus) *Saccharomyces cerevisiae* has been observed to degrade isomers of lindane more efficiently than other isomers following the order γ-HCH <β-HCH <α-HCH) [67,68]. In our study, biochar amendment caused a shift towards a number of soil bacterial genera that have been used around the world as bioremediation agents. In a study carried out by Saimmai et al [69], the genus *Chryseobacterium* was used to degrade oil hydrocarbons and resulted in 40.5% oil-degrading activity within 7 days of application. Select species of *Flavobacterium* have been used in batch reactions to completely degrade the organochlorine PCP (pentachlorophenol) at concentrations of 30 and 50 mg l^-1^ [70]. In our soil the proportion of these bacteria in the microbiological community was significantly increased as a result of biochar amendment, along with *Dyadobacter* which has the recorded ability to degrade a wide range of organic compounds and hydrocarbons in soil [71].

Although a shift in a number of genus and orders was observed under biochar amendment, the un-amended co-contaminated soil also contained phyla that are known to contain bioremediation agents. For example, *Chloroflexi*, genus *Luteibacter* and *Burkholderia* were represented in the microbial community and are known for their efficiency at degrading polychlorinated biphenyls (PCBs) and polycyclic aromatic hydrocarbons (PAHs). All have the natural ability to breakdown organochlorines such as HCH isomers and any increase in their activity may play a significant part in the degradation of these isomers. We propose that increased degradation of organochlorines observed for the biochar treatments was due to the activation of bacterial genus/species already present in the soil that are known for degradative mechanisms.

With respect to DDT degradation, a DDE:DDT ratio of 20:1 has been proposed by Elliott et al [72] to define a soil that has not received DDT input for a period of 20 years. A ratio lower than 0.2 suggests that the degradation of DDT to DDE may have been inhibited. A higher ratio therefore indicates active degradation of DDT in soil. In our study the DDE:DDT ratio increased to 0.25 as a function of 350°C biochar amendment to soil (Table 7). We propose that this increase in the DDE:DDT ratio is associated with a biochar-promoted increase in organochlorine-degrading microbial activity. The DDE:DDT ratio for the control soil was 0.16, and this was significantly increased (P<0.05) for three of the four biochar treatments (Table 7). The low DDE:DDT ratio for this contaminated soil suggests that DDT degradation has been historically inhibited, and we believe that this is a likely consequence of low microbial activity due to the presence of the co-contaminant As. The initial reduction in As concentration in soil solution (from 1.2 to 0.9 mg L^-1^ for the low temperature 2% amendment; DDE:DDT 0.25) may have alleviated As-related toxicity to microorganisms in the short term, allowing for a rapid increase in soil microbiological activity. A similar co-contaminant effect of Cu on DDT degradation was described by Gaw et al [73]. In this work, high Cu concentrations in orchard soils were assumed to have inhibited microbial pathways that transform DDT to DDE [10, 73].

**Table 7:** 4,4'-DDT and its breakdown derivative 4,4'-DDE (mg kg^-1^) under biochar treatments in co-contaminated soil after T = 180 d.

	DDE	DDT	DDE:DDT
**Control Soil**	0.50 ± 0.06^a^	3.13 ± 0.26^a^	0.16 ± 0.03^a^
**350°C BC Amended (1%)**	0.45 ± 0.01^a^	2.10 ± 0.06^b^	0.21 ± 0.00^b^
**350°C BC Amended (2%)**	0.43 ± 0.01^a^	1.72 ± 0.08^b^	0.25 ± 0.01^b^
**550°C BC Amended (1%)**	0.49 ± 0.01^a^	2.90 ± 0.32^a^	0.17 ± 0.02^a^
**550°C BC Amended (2%)**	0.47 ± 0.02^a^	2.17 ± 0.07^b^	0.22 ± 0.01^b^

A ratio between DDE:DDT in soil after treatment is also included. Significant errors are listed after the mean with all samples (mean *n* = 3; ± s.e.). Significant differences are noted by different letters.

### Practical relevance of this work

Co-contamination of soil with organic and inorganic chemicals at sheep dip sites is a long-lasting environmental issue. Our data show that 40 years after the cessation of dipping activities, the soil remains highly contaminated with As and a range of organochlorine compounds. Effective and simple remediation strategies for such land are therefore needed to manage or mitigate the risk associated with such contaminated land. Under controlled glasshouse conditions the use of willow biochar as a soil amendment led to an increase in soil microbial activity and ultimately a 25% reduction in DDT (and its products), a 75% decrease in lindane, and 80% reduction in alpha-HCH levels. Our results suggest that the use of biochar can change the composition and stimulate the activity of existing microbial communities within co-contaminated soils, leading to the degradation of certain organochlorines. The observed increase in DHA in soil amended with biochar, along with a change in bacterial community, provides evidence that these two factors play a major part in the degradation of organochlorines in soil. We believe that this degradation is catalysed by a short-term reduction in soluble co-contaminant concentration (As) which is effected by biochar amendment to soil. The microbial community that rapidly evolves as a result of this biochar effect is then able to withstand the re-release of As back into a bioavailable phase. More research is needed to understand the mechanisms behind this degradation and whether other organic amendments can induce similar changes. Future work will seek to investigate whether similar changes can be effected in the soil under field conditions.

## Supporting Information

S1 Fig Taxonomy summary for biological replicates following analysis through QIIME. (PDF)

S2 Fig Taxonomy analysis for treatment samples following pooling of biological replicates data. (PDF)

S3 Fig Shannon diversity collectors curves for samples following pooling of biological replicates. All curves reached saturated plateau phase. Blue line, Control sample; orange line, 350°C biochar; and red line, 550°C biochar.
(PDF)

S4 Fig Ordination plots derived from principal coordinates analyses of weighted unifrac distances between bacterial communities in the biological replicates of the Biochar treated and control samples. Blue, control samples; Red, 350°C biochar; and green, 550°C biochar.
(PDF)

S5 Fig Ordination plots derived from principal coordinates analyses of weighted unifrac distances between bacterial communities in the Biochar treated and control samples following combination of the biological replicates. Blue, control samples; Red, 350°C biochar; and green, 550°C biochar.
(PDF)
