# Supplementary material for: Biochar in Co-Contaminated Soil Manipulates Arsenic Solubility and Microbiological Community Structure, and Promotes Organochlorine Degradation
Source: PLoS One. 2015 Apr 29;10(4):e0125393. doi: 10.1371/journal.pone.0125393 (PMC4414470; doi:10.1371/journal.pone.0125393)
Supplement: S2 Fig — (PDF) [file pone.0125393.s002.pdf]

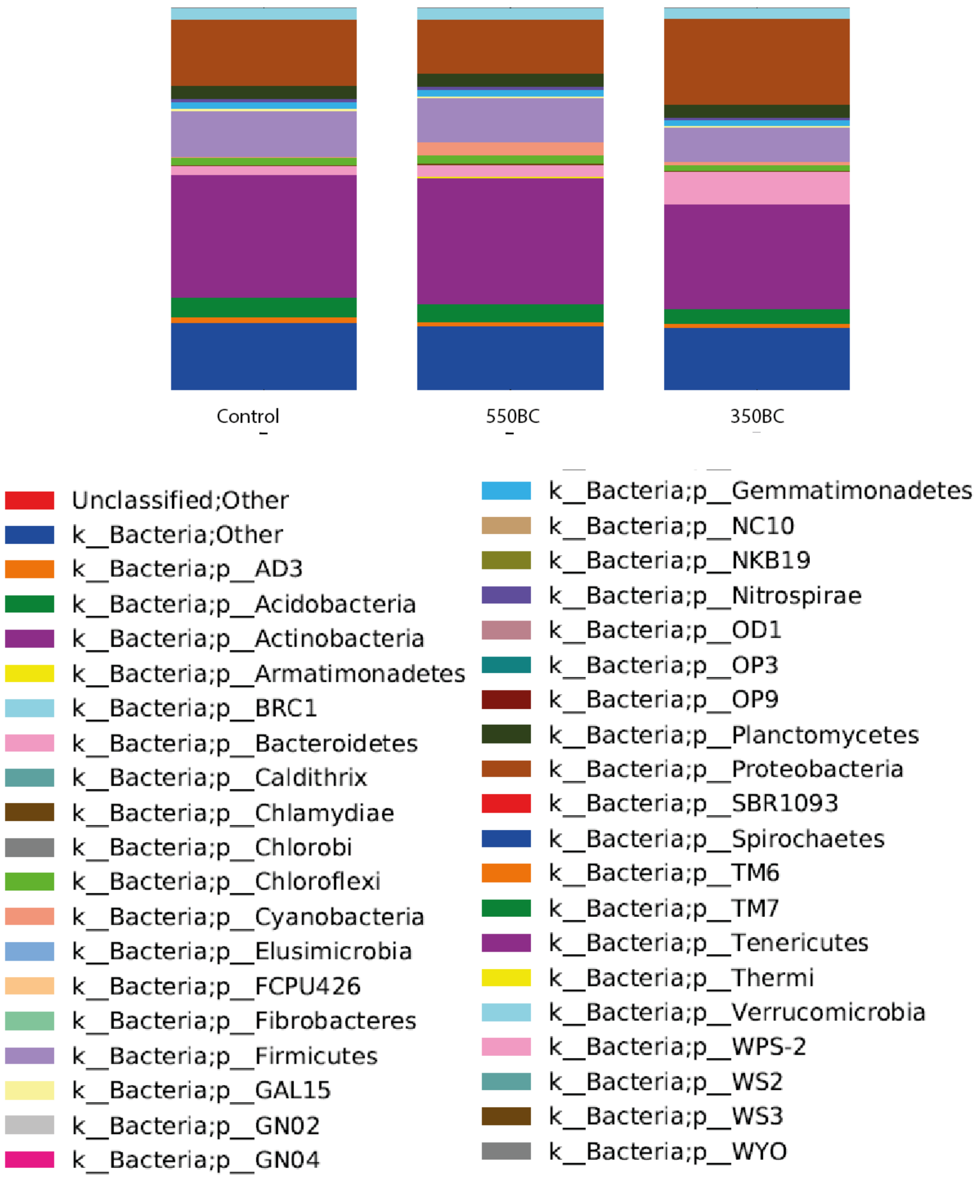

S2 Fig. Taxonomy analysis for treatment samples following pooling of biological replicates data.
