# Supplementary material for: Biochar in Co-Contaminated Soil Manipulates Arsenic Solubility and Microbiological Community Structure, and Promotes Organochlorine Degradation
Source: PLoS One. 2015 Apr 29;10(4):e0125393. doi: 10.1371/journal.pone.0125393 (PMC4414470; doi:10.1371/journal.pone.0125393)
Supplement: S3 Fig — All curves reached saturated plateau phase. Blue line, Control sample; orange line, 350°C biochar; and red line, 550°C biochar. (PDF) [file pone.0125393.s003.pdf]

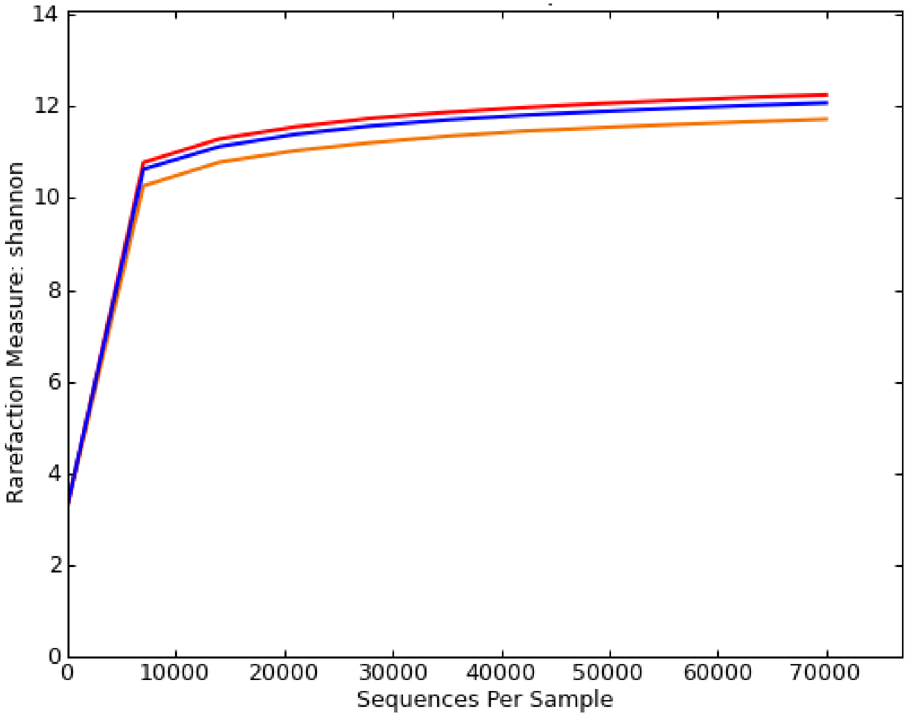

S3 Fig. Shannon diversity collectors curves for samples following pooling of biological replicates. All curves reached saturated plateau phase. Blue line, Control sample; orange line, 350°C biochar; and red line, 550°C biochar.
