# Supplementary material for: Biochar in Co-Contaminated Soil Manipulates Arsenic Solubility and Microbiological Community Structure, and Promotes Organochlorine Degradation
Source: PLoS One. 2015 Apr 29;10(4):e0125393. doi: 10.1371/journal.pone.0125393 (PMC4414470; doi:10.1371/journal.pone.0125393)
Supplement: S5 Fig — Blue, control samples; Red, 350°C biochar; and green, 550°C biochar. (PDF) [file pone.0125393.s005.pdf]

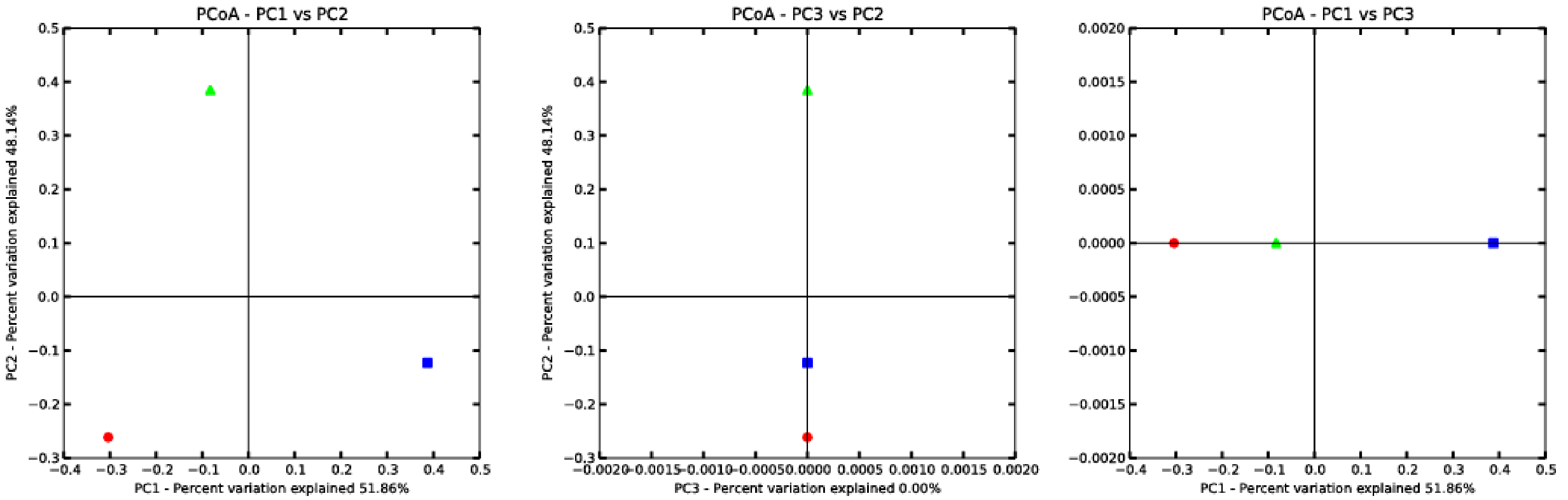

S5 Fig. Ordination plots derived from principal coordinates analyses of weighted unifrac distances between bacterial communities in the Biochar treated and control samples following combination of the biological replicates. Blue, control samples; Red, 350°C biochar; and green, 550°C biochar.
